# Lateral one-third gland resection in Cushing patients with failed adenoma identification leads to low remission rates: long-term observations from a small, single-center cohort

**DOI:** 10.1007/s00701-021-04830-2

**Published:** 2021-04-03

**Authors:** Lukas Andereggen, Luigi Mariani, Jürgen Beck, Robert H. Andres, Jan Gralla, Markus M. Luedi, Joachim Weis, Emanuel Christ

**Affiliations:** 1 Department of Neurosurgery, Neurocenter and Regenerative Neuroscience Cluster, Inselspital, Bern University Hospital, University of Bern, Bern, Switzerland; 2 Department of Neurosurgery, Kantonsspital Aarau, Aarau, Switzerland; 3 Department of Neurosurgery, University Hospital of Basel, Basel, Switzerland; 4 Department of Neurosurgery, Medical Center, University of Freiburg, Freiburg, Germany; 5 Department of Neuroradiology, Inselspital, Bern University Hospital, University of Bern, Bern, Switzerland; 6 Department of Anaesthesiology and Pain Medicine, Inselspital, Bern University Hospital, University of Bern, Bern, Switzerland; 7 Institute of Neuropathology, RWTH Aachen University Hospital, Aachen, Germany; 8 Department of Endocrinology, Diabetology and Metabolism, University Hospital of Basel, Basel, Switzerland

**Keywords:** Cushing’s disease, Remission, Adenoma, Petrosal sinus sampling, Pituitary surgery

## Abstract

**Background:**

Currently, there are no guidelines for neurosurgeons treating patients with Cushing’s disease (CD) when intraoperative adenoma identification is negative. Under these circumstances, a total hypophysectomy or hemi-hypophysectomy on the side indicated by inferior petrosal sinus sampling (IPSS) is the approach being used, although there is a subsequent risk of hypopituitarism. Data on whether one-third lateral pituitary gland resection results in cure of hypercortisolism and low rates of hypopituitarism remain inconclusive.

**Methods:**

Retrospective single-center study of CD patients with failed intraoperative adenoma identification and subsequent resection of the lateral one-third of the pituitary gland as predicted by IPSS. We assessed (i) histopathological findings, (ii) early and long-term remission rates, and (iii) rates of additional pituitary hormone insufficiency.

**Results:**

Ten women and three men met the inclusion criteria. At 3 months, remission was noted in six (46%) patients: three (23%) had histologically confirmed adenomas, two (15%) had ACTH hyperplasia, and one patient (8%) was positive for Crooke’s hyaline degeneration. New pituitary hormone deficits were noted in two patients (15%). After a median (±SD) follow-up of 14±4 years, recurrence was noted in two (15%) patients. Long-term control of hypercortisolism was attained by 10 patients (77%), with additional therapies required in nine (69%) of them.

**Conclusions:**

In CD patients with failed intraoperative adenoma visualization, lateral one-third gland resection resulted in low morbidity and long-term remission in 31% of patients without the need for additional therapies. Bearing in mind the sample size of this audit, the indication for lateral one-third-gland resection has to be critically appraised and discussed with the patients before surgery.

**Supplementary Information:**

The online version contains supplementary material available at 10.1007/s00701-021-04830-2.

## Introduction

Cushing’s disease (CD) is the most challenging disorder among hormone-secreting pituitary tumors [13]. CD is predominantly caused by a corticotroph pituitary microadenoma [62]. Despite modified protocols, not infrequently magnetic resonance imaging (MRI) fails to detect adrenocorticotropin-secreting (ACTH) pituitary microadenomas [20, 22, 65]. Thus, bilateral inferior petrosal sinus sampling (IPSS) remains the gold standard in identifying the source of Cushing’s syndrome (CS) in patients with negative or equivocal MRI findings [7, 10]. Nevertheless, subsequent intraoperative exploration still might reveal a pituitary gland with a normal appearance [7, 10]. Under these circumstances, no commonly accepted treatment guidelines exist, and hemi-hypophysectomy―or two-thirds gland resection―has been proposed as a valid option, with a subsequent risk of hypopituitarism [19]. Data remain inconclusive with regard to whether one-third resection of the pituitary gland on the side indicated by IPSS results in high remission rates and a low rate of additional pituitary hormone deficiencies.

In this audit of practice, we assessed (i) histopathological findings, (ii) early and long-term remission rates, and (iii) the rate of new pituitary hormone insufficiency in CD patients with negative MRI findings, failed intraoperative adenoma exploration, and subsequent lateral one-third gland resection on the side indicated by IPSS. Emphasis was placed on assessing the long-term outcome in these patients, given that high long-term recurrence rates are known [11, 49].

## Methods

### Study design

We performed a retrospective study evaluating data from our institutional database, which was prospectively maintained from October 1997 to January 2016. All consecutive patients met clinical and biochemical inclusion criteria for ACTH-dependent CS [27, 34, 64]. Bilateral IPSS was performed due to failed adenoma identification by pituitary MRI. Patients were included if the adenomas could not be identified intraoperatively after careful exploration of the entire gland at the time of transsphenoidal surgery (TSS). Thus, the lateral one-third of the pituitary gland was subsequently resected on the side indicated by IPSS. Tissue was sent to pathology for histological analysis. Early remission rates, hypopituitarism, and long-term outcome were extracted from the medical records.

### MR imaging and bilateral IPSS

PD/T2-weighted, unenhanced, contrast-enhanced, dynamic contrast-enhanced, and post contrast-enhanced overlapping images in three planes over the pituitary region of 1-mm fine-cut images were collected using a 1.5 Tesla MRI [6, 9, 10]. In patients with negative MRI and ACTH-dependent CS with equivocal responses to biochemical testing (i.e., ovine corticotropin-releasing hormone [oCRH] stimulation and/or high-dose dexamethasone suppression), bilateral IPSS was indicated [7, 10]. Details of this procedure have been described previously [7, 10]. In brief, both femoral veins were catheterized. A Hi-Flow Tracker 18 (Renegade Hi-Flow Microcatheter; Boston Scientific Target, Fremont, CA) was placed into each cavernous sinus to enable classification of the venous outflow on both sides. If the cavernous sinus could not be catheterized, the drainage type was recorded based upon an internal carotid artery injection. For venous blood sampling, the microcatheter was retracted distal to the cavernous sinus and placed into each proximal IPS. We determined the venous outflow variances on each side of the IPS after contrast injection and classified them as described by Shiu et al. [61] and Benndorf et al. [12]. An asymmetric IPS was defined as different venous outflow patterns of IPS seen when comparing the left and right sides.

As proposed by Oldfield et al., mean ACTH concentration of both the petrosal sinuses, divided by mean ACTH concentration of a peripheral vein, was indicative of a central ACTH source when gradients were ≥2.0 before and/or ≥3.0 after oCRH stimulation [47]. As for lateralization, mean ACTH concentration of one side of the petrosal sinuses divided by mean ACTH concentration of the other side was positive if gradients were ≥1.4 [46].

### Microsurgery

A transnasal transsphenoidal approach under microscopic magnification was used in all cases [28]. All operations were performed by the senior neurosurgeon (RWS).

**Fig. 1:**
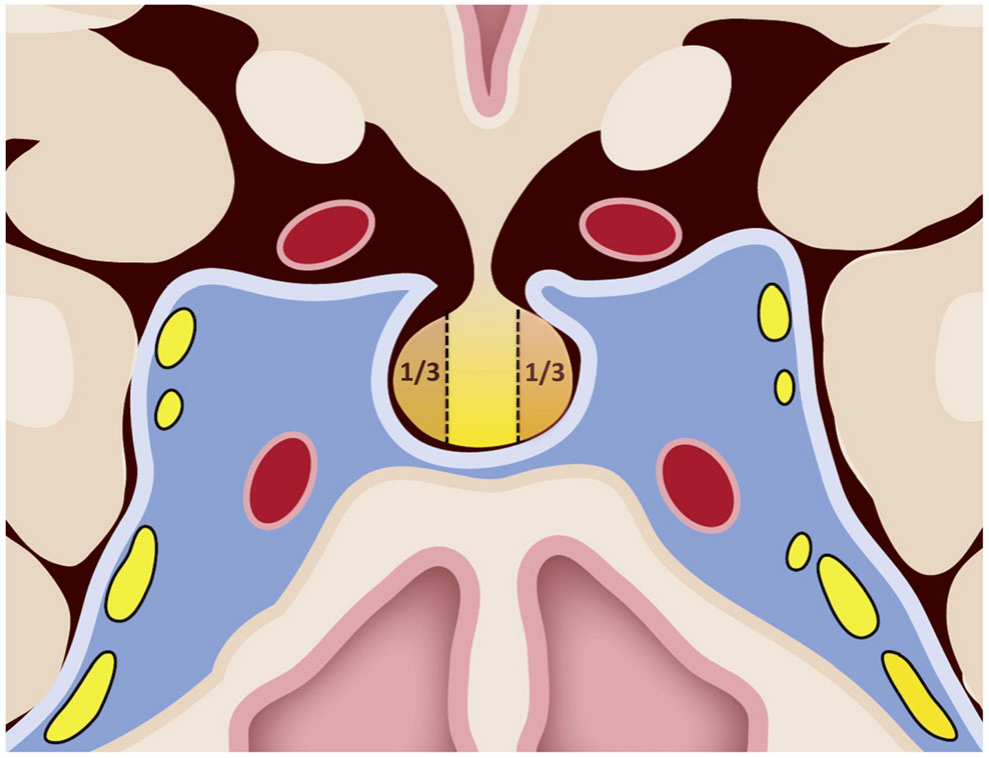
Coronal view of the pituitary gland and parasellar region with depiction of the surgical technique used. After an extensive opening of the sellar floor followed by cruciate dural opening, the peripheral gland was thoroughly explored and the lateral third (1/3) of the gland periphery was incised on the side indicated by IPSS if no adenoma was found. The tissue (a brownish-yellow color) of the lateral margins adjacent to the medical wall of the cavernous sinus was resected and sent to pathology

As microadenomas can induce limited bulging in the surface of the gland, we created a wide opening in the sellar floor to thoroughly expose the pituitary gland after extensive dural opening. The superior and inferior surfaces of the gland, as well as the lateral walls in direct contact with the medial wall of the cavernous sinus, were explored, and the lateral ligaments were divided, allowing for easier mobilization and inspection of the pituitary gland’s lateral surface [30]. If no bulging was noticeable, a vertical incision was made in the lateral gland on the side where the adenoma was suspected to be found, in order to explore the inside of the gland. Suspicious tissue was sent for frozen sectioning. If no adenoma was confirmed, a lateral one-third hypophysectomy was performed using pituitary rongeurs on the side indicated by IPSS (Fig. 1). This included the bottom of the gland in order to maximize the chance of microadenoma removal while minimizing new endocrinopathies [24, 30, 31, 60, 63]. Tissue was sent to pathology for histological analysis. To avoid rhinoliquorrhea, the surgical defect was filled with gelatin foam soaked in fibrin glue, and a synthetic absorbable vicryl patch was used to cover the sellar opening [3–5, 8, 10, 58].

### Histopathological analyses

Histopathological analyses were performed to confirm the presence of an ACTH-secreting adenoma. In brief, paraffin sections were stained for hematoxylin, reticulin, and ACTH. In addition, staining was performed for growth hormone, prolactin, thyroid-stimulating hormone, luteinizing hormone, and follicle-stimulating hormone. If no adenoma was found, the tissue was examined for ACTH hyperplasia or positive Crooke’s hyalinization as an indirect marker of hypercortisolism [48].

### Determination of remission rates

The early remission rate was determined 3 months postoperatively. Long-term remission rates were extracted ≥ 12 months postoperatively. Remission was defined as immediate postoperative serum cortisol <50 nmol/L and the need for a transitory cortisol replacement therapy. In addition to improvement in the clinical features of CS, remission was defined as normalization of 24 h urinary cortisol levels, normal salivary cortisol levels for three consecutive days, and/or a normal dexamethasone suppression test during the follow-up visit [27, 41, 64].

### Statistical analyses

Data were analyzed using IBM SPSS statistical software Version 24.0 (IBM Corp., New York, NY, USA) and GraphPad Prism (V7.04 Software, San Diego, CA, USA). Continuous variables were examined for homogeneity of variance and are expressed as mean ± standard deviation (SD) unless otherwise noted. For comparisons of means between the two groups, Student’s *t*-test was used for normally distributed data, and the Mann-Whitney test for nonparametric data. Categorical variables were compared using Pearson’s chi-square test or Fisher’s exact test, as appropriate. Differences were considered significant at *p* < 0.05 for reported two-sided *p* values.

## Results

### Patients’ characteristics

Ten women (77%) and three men (23%) met the study inclusion criteria. Patients’ characteristics are summarized in Table 1. Median age was 49 ± 12.4 years (range 24–63 years). All patients had negative MRI results and underwent IPSS, with the latter predicting a pituitary (central) lesion. Preoperative pituitary deficits were rare. Two patients (15%) showed isolated one-axis impairment (gonadotrophic deficiency in one patient and thyrotropic deficiency in another patient).

**Table 1:** Baseline characteristics and remission rates following lateral one-third-gland resection

Case No.	Age (yrs), sex	IPSS prediction	Histopathology	Early remission at 3 months	Long-term remission (months)	Additional therapy
1	53, m	Central left	Normal pituitary	No	Yes (203)	Bilateral adrenalectomy
2	41, f	Central right	ACTH hyperplasia	Yes	Yes (156)	None
3	49, f	Central left	Adenoma	No	Yes (180)	Bilateral adrenalectomy
4	40, f	Central right	Adenoma	Yes	Yes (228)	None
5	52, f	Central left	Adenoma	Yes	No (128)	Drug-treated
6	37, f	Central right	ACTH hyperplasia	Yes	No (168)	Gamma-knife surgery
7	50, f	Central right	Adenoma	Yes	Yes (171)	None
8	63, f	Central left	ACTH hyperplasia	No	Yes (170)	Drug-treated
9	55, m	Central right	ACTH hyperplasia	No	Yes (64)	None
10	60, f	Central right	pos. Crooke hyaline	Yes	Yes (24)	None
11	35, m	Central left	Adenoma	No	Yes (98)	Bilateral adrenalectomy
12	25, f	Central right	pos. Crooke hyaline	No	No (72)	Drug-treated
13	24, f	Central right	pos. Crooke hyaline	No	Yes (61)	Bilateral adrenalectomy

*No.*, number; *yrs*, years; *IPSS*, inferior petrosal sinus sampling; *m*, male; *f*, female; *ACTH*, adrenocorticotropic hormone; *pos.*, positive.

### Bilateral IPSS results

In Supplementary Table 1, classification of the venous outflow and documentation of the venous outflow symmetry are summarized. In addition, central to peripheral ACTH gradients and intersinus gradients (lateralization data) before and after oCRH stimulation are depicted.

In three patients, no data on the drainage pattern were available. Symmetric outflow was recorded in eight patients (62%) and asymmetric outflow in two patients (15%). A central-to-peripheral gradient ≥2.0 before oCRH stimulation was attained in 10 patients (77%), whereas a gradient ≥3.0 after oCRH stimulation was attained in all of them (100%). With regard to lateralization, normalized ACTH values (i.e., side-by-side gradients) were not significantly different between patients with a symmetric (7.3 ± 2.6) or an asymmetric outflow at baseline (18.2 ± 13.4; *p* = 0.19), but they were significantly different after oCRH stimulation (symmetric: 9.0 ± 4.9 versus asymmetric: 141.0 ± 93.9; *p* = 0.01).

We further noted that ACTH gradients were higher in patients with a histologically confirmed adenoma compared to those without, both before (19.2±8.7 vs. 6.6 ± 2.6; *p*=0.12) and after (74.8± 43 vs. 9.6 ± 5.5, *p*=0.08) oCRH stimulation, but not significantly. In addition, early remission rates were found not to be significantly different in patients with higher ACTH gradients compared to those with lower gradients, both before (12.2 ± 5 vs. 10.8 ± 6.2, *p*=0.87) and after (43.1 ± 38 vs. 27.4 ± 12.6; *p*=0.69) oCRH stimulation.

### Histopathological results

An ACTH-secreting adenoma was histologically confirmed in five patients (39%), an ACTH hyperplasia in four (31%), positive Crooke-hyaline in three patients (23%), and a normal pituitary gland in one patient (7%).

### Rates of remission

**Fig. 2:**
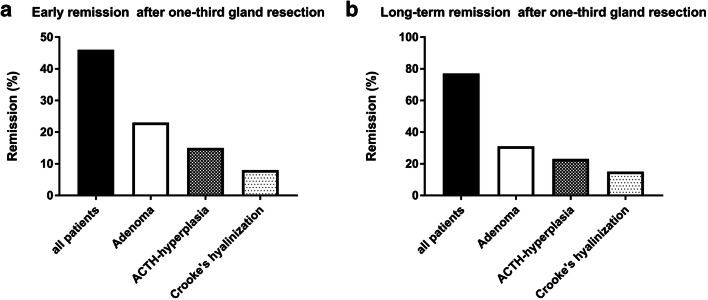
Early and long-term remission following lateral one-third-gland resection. At early follow-up, remission was noted in six patients (46%): three patients (23%) with histologically confirmed adenomas, two patients (15%) with ACTH hyperplasia, and one patient (8%) with positive Crooke’s hyaline degeneration (**a**). Long-term remission was noted in 10 patients (77%): four patients (31%) with initially confirmed adenoma, three patients (23%) with ACTH hyperplasia, two patients (15%) with positive Crooke-hyaline, and one patient (8%) with negative histology (**b**)

At 3 months, remission was noted in three patients (23%) with histologically confirmed adenomas, in two patients (15%) with ACTH hyperplasia, and in one patient (8%) with positive Crooke’s hyaline degeneration (Fig. 2a). When dichotomizing patients into those with confirmed adenoma and those without, we noted early remission in three patients (60%) with histologically confirmed adenoma vs. three (38%) without (*p*=0.59).

Recurrence occurred in three patients (25%) with initial cortisol normalization. For the long-term control of hypercortisolism, bilateral adrenalectomy was required in four patients (33%), metyrapone therapy in three (25%), and pituitary radiosurgery in one patient (8%) (Table 1).

After a median (±SD) follow-up of 14±4 years, long-term remission was noted in 10 patients (77%): four (31%) with initially confirmed adenoma, three (23%) with ACTH-hyperplasia, two (15%) with positive Crooke-hyaline, and one patient (8%) with negative histology (Fig. 2b).

### Complications of therapeutic interventions

No mortality due to the surgical intervention was noted. Postoperative complications consisted of rhinoliquorrhea in one patient―requiring temporary lumbar cerebrospinal fluid drain―and transient diabetes insipidus in another patient. New postoperative deficits requiring hormonal replacement were noted in two patients (15%), with testosterone replacement in one patient and additional growth hormone replacement in a patient with preoperative thyrotropic deficiency. We noted no vascular injuries, meningitis, or abscesses [57].

## Discussion

Our data show that in CD patients with failed intraoperative adenoma visualization, (i) lateral one-third gland resection resulted in low rates of additional pituitary insufficiencies, but also in low cure rates of hypercortisolism, and (ii) high long-term remission rates (>10 years) can be attained, but only with an interdisciplinary multimodal approach.

CD is the most challenging disorder among hormone-secreting pituitary tumors [42, 54]. This has been attributed to difficulty both in the diagnosis and treatment of primarily microadenomas, along with the accuracy of tests in the assessment of endogenous hypercortisolism [7]. TSS is considered the first-line treatment for CD, providing remission in 59–90% of patients in dedicated tertiary pituitary centers [33]. Thereby, a risk factor for persistent hypercortisolism has been attributed to unsuccessful intraoperative adenoma identification [19, 33, 51]. For these cases, total hypophysectomy or hemi-hypophysectomy has been proposed―but at the expense of hypopituitarism [55]. Our approach resulted in low morbidity and long-term cure in 31% of patients. Likewise, early remission has been described in 11 CD patients (38%) with negative adenoma identification and subsequent hemi-hypophysectomy [35]. In the other 29 patients (62%) of their cohort, additional treatments such as adrenalectomy or radiotherapy were required. However, the authors reported no specific morbidity or rates of associated hypopituitarism [35]. Instead, in patients with two-thirds pituitary gland resection, favorable long-term remission rates were reported in 18 (82%) of the 22 patients and new endocrine deficits requiring hormone replacement in 9% [19]. While we likewise noted low rates of pituitary insufficiency, we could not attain remission rates as high as those reported by Carr et al. when we limited the resection to the lateral one-third of the pituitary gland [35]. This is of interest given that the rate of histologically confirmed adenomas reported by Carr and colleagues was lower than we noted in our series (27% vs. 38% in our cohort) [19]. This finding might underscore the hypothesis that unsuccessful histological adenoma confirmation does not seem to be the only predictor of a low remission rate. In fact, failure of histologic confirmation of an ACTH source is an intriguing finding, as remission rates are reported even in the absence of histological adenoma confirmation [67]. Nevertheless, most findings report on a lower initial remission rate and a higher long-term recurrence rate in patients without histological evidence of an adenoma [51]. On the other hand, the higher remission rates reported by Carr et al. might be due to removal of more pituitary tissue, thus increasing the likelihood of removing parts of the adenoma [39]. It is thus conceivable that removing adenohypophyseal corticotroph tissue in the central region of the gland known as the mucoid wedge might increase the chance for long-term cure [39]. Comtois and colleagues reported on 11 patients with absent intraoperative adenoma visualization, in whom a partial hypophysectomy of the central mucoid wedge was performed. This resulted in some remission but significantly lower cure rates as compared to those patients with intraoperative adenoma identification [25]. However, Carr and colleagues also reported that unlike Comtois’ observation [25], in their extensive experience with CD microadenomas, tumors were more commonly located in the lateral gland [19]. This underlines the finding that ACTH adenomas were localized in the lateral aspect of the pituitary gland in the majority of cases [37]. Likewise, bilateral IPS sampling in Cushing patients confirms a lateralization in the majority of cases [19, 37]. In the rare event of negative intraoperative adenoma visualization, the possibility of ectopic ACTH secretion has yet to be considered, despite high sensitivity and specificity attributed to bilateral IPSS [7, 10]. There have been several attempts to improve the predictive value of the intersinus ACTH gradient. In 2012, Mulligan and colleagues demonstrated improved adenoma lateralization using a prolactin-adjusted intersinus ACTH gradient of > 1.4 in their series [44]. Namely, IPSS prolactin can help to confirm correct catheter placement during venous sampling, thus improving differentiation between CD and EAS in the absence of proper IPS venous efflux [26, 50, 53]. However, further evaluation of the prolactin-adjusted ACTH ratio is needed to prove reliable surgical guidance [50]. Also, it has been suggested that anatomical variation like intersinus communication might explain sampling failures [53]. In our previous work, we noted that IPS asymmetry did not diminish the prediction of the adenoma side [8]. However, the small sample size of this cohort precludes us from undertaking statistical analysis to compare outcome with regard to outflow symmetry. In addition, we have to keep in mind that surgeon experience is of particular importance to guarantee a favorable outcome in the treatment of CD [17, 36].

Given that CD is most commonly caused by a microadenoma, tissue for pathologic diagnosis using standard techniques might be very limited during surgical exploration [29]. While we sent suspicious tissue for frozen sectioning and proceeded with lateral one third hypophysectomy in the case of a negative result, it must be taken into consideration that the reliability of frozen results varies considerably. It has been shown that the addition of intraoperative cytological analyses during surgery provides a useful armamentarium in the diagnosis of CD [52]. In fact, in some cases, the presence of an adenoma can only be proven by cytological preparation, which improves the adenoma identification rate and surgical outcome compared to classical immunostaining methods [38].

Given the similar remission rates attained when limiting the approach to the lateral two-thirds compared to hemi-hypophysectomy or subtotal hypophysectomy, subtotal resection might be preferred with regard to postoperative pituitary hormone insufficiency, although results remain ambiguous [17, 19]. As for ACTH-producing adenomas, the risk of postoperative pituitary dysfunction can be higher than for other types of pituitary adenomas, given the need for more extensive intraoperative dissection of the pituitary gland, even in the case of small adenomas [56]. Subtotal hypophysectomy results in new hypopituitarism, with incidence rates ranging from 1 to 35% [23, 33, 35]. Interestingly, Rees and colleagues noted that the extent of surgical exploration predicted the development of hypopituitarism (88% total hypophysectomy, 33% hemi-hypophysectomy, 14% selective adenomectomy), but not remission (75% total hypophysectomy, 87% hemi-hypophysectomy, 71% selective adenomectomy) in CD patients [55]. More surprisingly, Yamada et al. reported that none of their 10 patients with hemi-hypophysectomy was noted to have hypopituitarism requiring hormonal replacement [66]. While hormonal insufficiencies vary considerably among studies and are inconsistently reported, it is conceivable that a limited resection results in lower incidences of hormonal disorders following surgery. Thus, our long-term cure rates in 31% of patients without the need for additional therapies might still be acceptable for the individual patient with regard to the low incidence of new hormonal disorders. However, with a follow-up in excess of 10 years, we noted that high remission rates could be attained when interdisciplinary multimodal therapies were combined with an initial surgical approach. How to treat individual patients after unsuccessful remission should be discussed by neuroendocrinologists and pituitary surgeons in order to develop a proper strategy. We noted the use of metyrapone therapy in up to 25% of patients for the long-term control of hypercortisolism, an effective approach confirmed by a recent meta-analysis [15]. Alternatively, repeat surgery can be a safe next step prior to radiation or medical therapy in CD patients [18, 59].

These options are mainly indicated in patients with MR-proven sellar lesions, however. In addition, bilateral adrenalectomy―which was required in no less than 33% of patients in our cohort―has been described as an important option in CD, especially when other treatment options have failed over time [21, 43]. Still, whether negative histology should trigger a different approach with regard to multimodal treatment remains unclear.

To sum up, limiting pituitary gland resection to the lateral one-third is a safe approach with favorable outcome in the face of new endocrine deficits, but remission rates are low.

## Study limitations

The number of patients in our study is relatively small, and statistical uncertainty owing to this small sample size precluded us from undertaking statistical analysis to compare outcome within the subgroups (i.e., adenoma, ACTH hyperplasia, or positive Crooke-hyaline), or with regard to outflow symmetry encountered during IPS. However, it offers reliable insight derived from long-term data obtained over two decades with a follow-up of 14 years.

In contrast to microscopic TSS, an endoscopic approach might be associated with higher gross total tumor removal [40], but results remain ambiguous [2]. In the treatment of CD, there seem to be no clear advantages of a purely endoscopic approach in terms of clinical outcome [1, 16, 45]. In particular, endoscopic surgery for patients with CD has been shown to have comparable results for microadenomas, with presumably better results for macroadenomas [14], which were not represented in the cohort presented here.

Given the long-term follow-up with recruitment of patients starting as early as 1997, patients were screened with a 1.5 Tesla MRI for the depiction of a microadenoma [6]. While some authors support the use of contrast-enhanced 3 Tesla MRI for CD, most of these studies comparing the detection rate of the pituitary 3 Tesla and 1.5 Tesla MRI are using different imaging protocols, thus limiting the diagnostic accuracy of high-field-strength MRI in patients with CD [65]. In particular, a higher magnetic field might not be advantageous in the diagnosis of microadenomas per se [32, 65].

## Conclusions

In CD patients with failed intraoperative adenoma visualization, lateral one-third gland resection resulted in low morbidity and long-term remission in 31% of patients without the need for additional therapies. Bearing in mind the sample size of this audit, the indication for lateral one-third-gland resection has to be critically appraised and discussed with the patients before surgery.

## Supplementary Information

Supplementary Table 1: Radiological characteristics of patients with bilateral IPSS (DOCX 19 kb).
