# Supplementary material for: Lateral one-third gland resection in Cushing patients with failed adenoma identification leads to low remission rates: long-term observations from a small, single-center cohort
Source: Acta Neurochir (Wien). 2021 Apr 3;163(11):3161–9. doi: 10.1007/s00701-021-04830-2 (PMC8520517; doi:10.1007/s00701-021-04830-2)
Supplement: Supplementary file 1 — Radiological characteristics of patients with bilateral IPSS (DOCX 19 kb). [file 701_2021_4830_MOESM1_ESM.docx]

**Supplementary Table 1: Radiological characteristics of patients with bilateral IPPS**

| Case No. | Centr./peri. before/after CRH | Lateral before/after CRH | Centr./peri  before/after CRH | Lateral  before/after CRH | BIPSS prediction | Venous  drainage r/l | Drainage  pattern |
| --- | --- | --- | --- | --- | --- | --- | --- |
| 1 | 33.1 / 85.9 | 7.3 / 1.5 | +/+ | +/+ | Central left | I/I | Symmetric |
| 2 | 1.6 / 3.9 | 1.5/ 1.7 | –/+ | +/+ | Central right | III/III | Symmetric |
| 3 | 21.9 / 51.9 | 47.0 / 88.7 | +/+ | +/+ | Central left | * |  |
| 4 | 1.9 / 3.4 | 2.4 / 4.9 | –/+ | +/+ | Central right | * |  |
| 5 | 50.1 / 149. 2 | 4.6 / 3.2 | +/+ | +/+ | Central left | * |  |
| 6 | 16.3 / 73.5 | 10.5 /3.4 | +/+ | +/+ | Central right | I/I | Symmetric |
| 7 | 13.7 / 195.2 | 31.6 / 234.9 | +/+ | +/+ | Central right | V/I | Asymmetric |
| 8 | 1.4 / 6.6 | 1.6 / 5.2 | –/+ | +/+ | Central left | III/III | Symmetric |
| 9 | 3.2 / 215.9 | 4.8 / 46.9 | +/+ | +/+ | Central right | III/I | Asymmetric |
| 10 | 7.8 / 11.5 | 22.6 / 10.8 | +/+ | +/+ | Central right | I/I | Symmetric |
| 11 | 4. 9 / 11.4 | 10.2 / 42.4 | +/+ | +/+ | Central left | I/I | Symmetric |
| 12 | 24.8 / 46.6 | 1.2 /1.5 | +/+ | –/+ | Central right | I/I | Symmetric |
| 13 | 11.7 / 22.8 | 3.2 / 6.3 | +/+ | +/+ | Central right | III/III | Symmetric |

No., number; centr, central; peri. Peripheral; BIPSS, bilateral inferior petrosal sinus sampling; oCRH, ovine corticotropin-releasing hormone; r, right; l, left; * information on the venous drainage pattern was not available
